# COVID-19 Student Stress Questionnaire: Development and Validation of a Questionnaire to Evaluate Students’ Stressors Related to the Coronavirus Pandemic Lockdown

**DOI:** 10.3389/fpsyg.2020.576758

**Published:** 2020-10-22

**Authors:** Maria Clelia Zurlo, Maria Francesca Cattaneo Della Volta, Federica Vallone

**Affiliations:** ^1^Dynamic Psychology Laboratory, Department of Political Sciences, University of Naples Federico II, Naples, Italy; ^2^Department of Humanities, University of Naples Federico II, Naples, Italy

**Keywords:** COVID-19, health psychology, pandemic lockdown, university students, validation

## Abstract

Clinical observations suggest that during times of COVID-19 pandemic lockdown university students exhibit stress-related responses to fear of contagion and to limitations of personal and relational life. The study aims to describe the development and validation of the 7-item COVID-19 Student Stress Questionnaire (CSSQ), a measurement tool to assess COVID-19-related sources of stress among university students. The CSSQ was developed and validated with 514 Italian university students. Exploratory Factor Analysis (EFA) was conducted with one split-half sub-sample to investigate the underlining dimensional structure, suggesting a three-component solution, which was confirmed by the Confirmatory Factor Analysis (CFA) with the second one split-half sub-sample (CFI = 0.95; TLI = 0.95; RMSEA = 0.06). The CSSQ three subscales measure COVID-19 students’ stressors related to (1) Relationships and Academic Life (i.e., relationships with relatives, colleagues, professors, and academic studying); (2) Isolation (i.e., social isolation and couple’s relationship, intimacy and sexual life); (3) Fear of Contagion. A Global Stress score was also provided. The questionnaire revealed a satisfactory internal consistency (Cronbach’s alpha = 0.71; McDonald’s omega = 0.71). Evidence was also provided for convergent and discriminant validity. The study provided a brief, valid and reliable measure to assess perceived stress to be used for understanding the impact of the COVID-19 pandemic lockdown among university students and for developing tailored interventions fostering their wellbeing.

## Introduction

The Coronavirus Disease 2019 (COVID-19) has been defined as an extreme health, economic and social emergency and it was declared a global pandemic by the World Health Organization on March 2020 (World Health Organization, 2020), resulting in lockdown and life restrictions in Italy as worldwide in the attempt to prevent and slow the spread of the virus.

Comparable previous emergencies, such as the SARS outbreak, were strongly demonstrated as spreading stress and inducing psychological disease in terms of depression, anxiety but also panic attacks, and even psychotic symptoms, delirium, and increased rates of suicidal (Xiang et al., 2020). These results have been recently confirmed with respect to the current COVID-19 pandemic (Brooks et al., 2020; Zandifar and Badrfam, 2020), particularly in terms of high levels of psychological distress (Qiu et al., 2020), depression (Wang et al., 2020), anxiety (Horesh and Brown, 2020; Lima et al., 2020; Rajkumar, 2020), fear and panic behaviors (Shigemura et al., 2020).

In this perspective, a review conducted by Brooks et al. (2020) on the psychological impact of quarantine periods and outbreak confinements in last decades (e.g., the SARS outbreak, the 2009 and 2010 H1N1 influenza pandemic) identified specific common experiences such as fear of contagion, fear and frustration related to inadequate supplies (e.g., basic necessities and medical supplies), sense of confusion due to inadequate quality of information from public health authorities, sense of isolation, frustration and boredom due to loss of usual routine and to reduced social contacts (Brooks et al., 2020).

Furthermore, the COVID-19-related containment measures imposed massive work and school closures, segregation and social distancing, deeply impacting on personal and relational life and exposing people to experience uncertainty, feelings of isolation, and sense of “losses” in terms of motivation, meaning, and self-worth (Williams et al., 2020).

In view of that, research made several efforts to better explore the psychological impact of the ongoing Coronavirus global outbreak, developing and validating specific tools.

In particular, the Fear of COVID-19 Scale (FCV-19S; Ahorsu et al., 2020; Soraci et al., 2020) and the Coronavirus Anxiety Scale (CAS; Lee, 2020a) were developed to assess, respectively, perceived COVID-related fear and anxiety. Moreover, the COVID-19 Peritraumatic Distress Index (CPDI; Costantini and Mazzotti, 2020; Qiu et al., 2020) was developed to assess the frequency of anxiety, depression, specific phobias, cognitive change, avoidance and compulsive behavior, physical symptoms and loss of social functioning.

Finally, the COVID-19 Stress Scales (CSS; Taylor et al., 2020) was developed to measure the psychological impact of COVID-19 in terms of danger and contamination fears, fears about economic consequences, xenophobia, compulsive checking and reassurance seeking, and traumatic stress symptoms.

Overall, the instruments reported above specifically addressed the impact of the COVID-19 outbreak in terms of psychological outcomes, without addressing and identifying specific sources of stress related to relational and daily life changes induced by the COVID-19 pandemic lockdown. Indeed, the COVID-19 pandemic-related experiences induced not only fears of contagion and social isolation but also significant modifications in several aspects of daily routine, mainly influencing (hindering or intensifying) all relationships, such as those with relatives, with the partner, with friends, with colleagues. Consequently, it emerged the need to develop instruments able to address not only the potential effects of isolation and fear of contagion but also of modifications of all significant relationships in daily life, so considering all potentially perceived sources of stress featuring the experience of pandemic lockdown.

Furthermore, in line with the transactional perspective (Lazarus and Folkman, 1984), stress is considered a dynamic relational process, which depends on the constant interplay between individual factors (e.g., age, gender) and situational factors, so requiring to take into account specificities of target populations when defining tools to evaluate perceived sources of pressure.

From this perspective, the academic context was deeply affected by the lockdown restrictions worldwide. Indeed, due to the massive closure of colleges and universities (United Nations Educational, Scientific and Cultural Organization, 2020), all the scheduled activities and events were postponed/annulled, campuses and students’ accommodations were forced to evacuations, all the formal and informal interactions were shifted to online platforms, leading to a substantial change in students’ customary life.

Different studies exploring factors associated to COVID-19 outbreak among university students highlighted high levels of anxiety and worries about academic delays and influence of the epidemic on daily life, due to the disruption in students’ daily routine, in terms of activities, objectives and social relationships (Cao et al., 2020; Chen et al., 2020; Lee, 2020b; Sahu, 2020). Indeed, the quarantine hindered the possibility to experience the university life, impacting on academic studying (i.e., uncertainties related to annulment/delays of activities, difficulties in employment of online platforms for the distance learning), but also impairing the possibility to benefit from the relationships that may represent anchor in students’ life, such as those with peers, colleagues, and professors (Lee, 2020b; Sahu, 2020). In addition, also considering the increasingly key role played by romantic relationships in the young population (Anniko et al., 2019), research also outlined the potential changes in couple’ relationship, intimacy, and sexual life due to the COVID-19 pandemic (Li et al., 2020; Rosenberg et al., 2020).

Moreover, whether, on the one hand, the abovementioned relationships with partner, friends, peers, colleagues, and professors were subject to a radical reduction and standstill, on the one other hand, in most of the cases, relationships with relatives were deeply intensified. Indeed, the majority of students were forced to return back home, also resulting from the campus dormitory evacuations, inducing an increased exclusivity of interaction with relatives, potentially exacerbating frustration and conflicts. This particularly when considering students living in already disadvantaged conditions and/or suffering from abusive home experiences (Lee, 2020b).

Overall, whether it’s clear that university students’ life was subject to broad modifications, up to date, there are no specific tools to understand, comprehensively identify and assess specific sources of stress featuring university students’ COVID-19-related experiences. This, however, could help in early recognize those students at higher risk for developing a significant psychological disease related to the pandemic lockdown, and, accordingly, provide timely and tailored interventions fostering their wellbeing.

Responding to this need, the present study aimed at proposing and validating a newly developed measurement tool to specifically assess sources of stress related to the COVID-19 pandemic lockdown among university students, namely the COVID-19 Student Stress Questionnaire (CSSQ).

Seven potential sources of stress have been hypothesized and operationalized. These sources have been defined as connected not only to fear of contagion and to experience of isolation but also to the potential abovementioned changes in students’ daily life and routine. In particular, it was hypothesized that induced changes in academic studying and relationships with friends, partner, university colleagues, professors and relatives could constitute significant perceived COVID-19 pandemic lockdown-related sources of stress among university students.

Hypotheses and research questions to rigorously check the validity and reliability of the COVID-19 Student Stress Questionnaire (CSSQ) are listed in Table 1.

**TABLE 1:** Research questions and hypotheses of the validation study.

Level of evidence and reliability	Number of research questions (R) or hypothesis (H)	Research question or hypothesis
Evidence based on construct validity	R1	Are all the items of the proposed COVID-19 Student Stress Questionnaire (CSSQ) relevant and appropriate in terms of the construct of COVID-19-related perceived stress among university students?
	R2	Is the CSSQ a uni-dimensional or multidimensional measure?
	H1	The data from this study reveal correlations, so that significant and coherent factors can be identified.
	H2	A factorial structure of the CSSQ can be confirmed.
Evidence based on convergent validity	H3	The standardized factor loadings, and the values of Composite Reliability and Average Variance Extracted of all factors are adequate.
	H4	There are moderate to strong correlations between the scales scores of the CSSQ and the standardized scales scores of the SCL-90-R.
Evidence based on discriminant validity	H5	The square root of the Average Variance Extracted of factors is above the correlations among the factors of the CSSQ.
	H6	There are moderate correlations among the CSSQ subscales scores, and strong correlations between the CSSQ subscales scores and the Global Stress score.
Reliability: internal consistency	H7	The CSSQ shows satisfactory internal consistency.

## Materials and Methods

### Participants and Sampling

Online survey data were collected from 15 April to 15 May 2020 with students from the University of Naples Federico II. This period fully corresponded to the pandemic lockdown due to COVID-19 in Italy, and students were experiencing the consequences of university closures, with massive social restrictions. The participants were recruited through Microsoft Teams. Students were contacted and given all the information about the study, and they were asked their participation on a voluntary basis. All the participants were fully informed about the aims of the study and about the confidentiality of the data, and they were also assured that the data would be used only for the purpose of the research and refusal to participate would not affect their current and future course of study in any way. The study was approved by the Ethical Committee of Psychological Research of the University where the study took place (IRB:12/2020). Research was performed in accordance with the 1964 Helsinki declaration and its later amendments or comparable ethical standards. Informed consent was obtained from each student prior to participating in the study. Every precaution was taken to protect the privacy of research subjects and the confidentiality of their personal information. Overall, 514 university students voluntarily enrolled in the study and completed online Microsoft Teams forms.

### Measures

The questionnaire included a section dealing with background information (i.e., Gender, Age, Degree Program, Year of study), the proposed 7-item COVID-19 Student Stress Questionnaire, and a measure for psychophysical health conditions.

#### COVID-19 Related Sources of Stress Among University Students

The COVID-19 Student Stress Questionnaire (CSSQ) was specifically developed to assess university students’ perceived stress during the COVID-19 pandemic lockdown. It consists of 7 items on a 5-point Likert scale ranging from zero (“Not at all stressful”) to four (“Extremely stressful”). For the purpose of instrument design, perceived stress was operationalized based on transactional models of stress (Lazarus and Folkman, 1984). Each item was developed to cover different domains that could have been subject to variations due to the COVID-19 pandemic lockdown, and, therefore, that may be potentially perceived as sources of stress (i.e., risk of contagion; social isolation; relationship with relatives; relationship with colleagues; relationship with professors; academic studying; couple’s relationship, intimacy and sexual life). The scale provides a Global Stress score ranging from 0 to 28.

#### Psychophysical Health Conditions

The Symptom Checklist-90-Revised (SCL-90-R; Derogatis, 1994; Prunas et al., 2010) was used to assess self-reported psychophysical health conditions. The scale comprises 90 items on a 5-point Likert scale ranging from zero (“Not at all”) to four (“Extremely”) and divided into nine subscales: Anxiety (10 items, Cronbach’s α = 0.84), Depression (13 items, Cronbach’s α = 0.87), Somatization (12 items, Cronbach’s α = 0.83), Interpersonal Sensitivity (9 items, Cronbach’s α = 0.83), Hostility (6 items, Cronbach’s α = 0.80), Obsessive-Compulsive (10 items, Cronbach’s α = 0.82), Phobic Anxiety (7 items, Cronbach’s α = 0.68), Psychoticism (10 items, Cronbach’s α = 0.77), and Paranoid Ideation (6 items, Cronbach’s α = 0.76). Participants were asked to indicate how much these problems have affected them during the past 4 weeks (e.g., Anxiety subscale: “Tense or keyed up”, “Fearful”; Depression subscale: “Hopeless about future”, “No interest in things”). The scale also provides a global index, namely the Global Severity Index (GSI). GSI is the sum of all responses divided by 90, and it indicates both the number of symptoms and the intensity of the disease (GSI Cronbach’s α = 0.97).

### Data Analysis

For the validity testing of the CSSQ we used the European Federation of Psychologists’ Association’s (EFPA) standards and guidelines (Evers et al., 2013), which describe the standard method for validity testing by the following levels of evidence: 1) Construct validity; 2) Criterion validity: (a) Post-dictive or retrospective validity; (b) Convergent validity; (c) Discriminant validity. In the present study, validity evidence was examined in relation to Construct validity, Convergent validity, and Discriminant validity.

#### Evidence Based on Construct Validity

Evidence based on construct validity was examined to answer research questions 1 and 2 and to test hypotheses 1 and 2 (Table 1). To examine the validity of the COVID-19 Student Stress Questionnaire (CSSQ) we used a two-step analytic strategy. First, the entire study sample (*N* = 514) was split using a computer-generated random seed. According to the rules of thumb for sample size in factor analysis, the sample size for each sub-sample (*n* = 257) was considered adequate to explore the structure of the 7-item CSSQ (Comrey and Lee, 1992; Costello and Osborne, 2005; DeVellis, 2017). Construct validity was analyzed using Exploratory Factor Analysis (EFA) and Confirmatory Factor Analysis (CFA).

EFA was performed in the first split-half (Sub-sample A, *n* = 257) to explore the latent dimensional structure (R1 and R2) and to identify significant and coherent factors (H1). Principal Components Analysis (PCA) with oblique promax rotation was used. The choice of non-orthogonal rotation was justified on the hypothesis that the factors would be correlated. The factorability of the correlation matrix of the scale was evaluated by Kaiser–Meyer–Olkin (KMO) measure and Barlett test of sphericity. Criteria for extraction and interpretation of factors were as follows: eigenvalues > 1.0, Cattell’s scree test and inspection of scree plot, communality ≥ 0.30 for each item and factor loading > 0.32 for each item loading on each factor (Costello and Osborne, 2005).

CFA was performed in the second split-half sub-sample (Sub-sample B, *n* = 257) to determine the goodness-of-fit of the extracted factor model (H2). Standard goodness-of-fit indices were selected *a priori* to assess the measurement models: χ^2^ non-significant (*p* > 0.05), Tucker-Lewis Index (TLI > 0.95), Root Mean Square Error of Approximation (RMSEA < 0.08) and Comparative Fit Index (CFI > 0.95) (Hu and Bentler, 1998).

#### Evidence Based on Convergent Validity

Evidence based on convergent validity was explored to test hypotheses 3 and 4 (Table 1). Convergent validity was tested, first, by calculating standardized factor loadings, composite reliability (CR), and average variance extracted (AVE) of factors (H3). If the standardized factor loadings of a questionnaire are > 0.5 and statistically significant, and the values of CR and AVE of each factor are higher than 0.7 and 0.5, respectively, the questionnaire is considered as having a satisfactory convergent validity (Fornell and Larcker, 1981; Hair et al., 2010). Moreover, convergent validity was assessed by correlational analyses (Pearson’s correlation coefficient) between the scales scores of the newly developed COVID-19 Student Stress Questionnaire and the standardized scales scores of the SCL-90-R (nine subscales and Global Severity Index) (H4). The effects size were interpreted following Cohen’s thresholds (*r* < 0.30 represents a weak or small correlation; 0.30 < *r* < 0.50 represents a moderate or medium correlation; *r* > 0.50 represents a strong or large correlation) (Cohen, 1988).

#### Evidence Based on Discriminant Validity

Evidence based on discriminant validity was explored to test hypotheses 5 and 6 (Table 1). Discriminant validity was evaluated by comparing the square root of the average variance extracted (SQRT AVE) with the correlations between latent constructs (H5). When the SQRT AVE is above the correlations among factors, a questionnaire is considered as having an acceptable discriminant validity (Fornell and Larcker, 1981). Furthermore, discriminant validity was also tested basing on the correlations between the CSSQ subscales and the Global Stress scores using the Pearson’s correlation coefficient (H6).

#### Evidence Based on Internal Consistency

Evidence based on internal consistency was explored to test hypothesis 7 (Table 1). Item means, standard deviations, and mean inter-item correlation (between 0.15 and 0.50) were evaluated (Clark and Watson, 1995). Moreover, for the reliability test, Cronbach’s Alpha (Cronbach, 1951) and McDonald’s Omega (McDonald, 1999) were used to assess the internal consistency of the questionnaire, considering α ≥ 0.70 (Santos, 1999) and ω ≥ 0.70 (McDonald, 1999) as indices of satisfactory internal consistency reliability (H7).

Finally, means, standard deviations, and ranges of the newly developed COVID-19 Student Stress Questionnaire (CSSQ) scales were calculated.

## Results

### Characteristics of Participants

Characteristics of the total sample (*N* = 514) as well as of each sub-sample (A and B) are shown in Table 2. The total sample consisted of 372 women and 142 men, with a combined mean age of 19.92 (*SD* = 1.50) years. The sample was composed of students enrolled in Philosophy (*n* = 10, 1.9%), Modern Languages and Literature (*n* = 44, 8.6%) and Psychology (*n* = 460, 89.5%) degree programs; the majority of them were 1st year students (1st year *n* = 400, 77.8%; 2nd year *n* = 46, 8.9%; 3rd year *n* = 68, 13.3%).

**TABLE 2:** Characteristics of study participants.

	Sub-sample A *n* = 257		Sub-sample B *n* = 257		Total Sample *N* = 514	
			
Characteristics	Value	Range	Value	Range	Value	Range
**Gender [*n* (%)]**						
Male	69 (26.8)		73 (28.4)		142 (27.6)	
Female	188 (73.2)		184 (71.6)		372 (72.4)	
**Age [*Mean (*SD*)]***	19.95 (1.56)	[18–26]	19.92 (1.43)	[18–26]	19.92 (1.50)	[18–26]
**Degree Program [*n* (%)]**						
Philosophy	6 (2.3)		4 (1.6)		10 (1.9)	
Modern Languages and Literature	23 (8.9)		21 (8.2)		44 (8.6)	
Psychology	228 (88.8)		232 (90.2)		460 (89.5)	
**Year of Study [*n* (%)]**						
1st year	197 (76.7)		203 (79.0)		400 (77.8)	
2nd year	27 (10.5)		19 (7.4)		46 (8.9)	
3rd year	33 (12.8)		35 (13.6)		68 (13.3)	

### Construct Validity

Construct validity (research question 1) was examined by conducting EFA and CFA.

#### Exploratory Factor Analysis

Exploratory Factor Analysis (EFA) using Principal Components Analysis (PCA) with oblique promax rotation was carried out to investigate the underlining dimensional structure of the CSSQ. The assessment of factorability showed that the Kaiser–Meyer–Olkin measure was 0.73 and Bartlett’s test of sphericity was significant (χ*^2^* = 332.26, df = 21, *p* < 0.001) indicating that the data were adequate for the factor analysis, supporting hypothesis 1. The examination of the scree plot produced a departure from linearity corresponding to a three-component result; the scree-test also confirmed that our data should be analyzed for three components, responding to research question two. The first three eigenvalues were 2.61, 1.20, and 1.00. The three-component solution explained a variance of 67.09% from a total of 7 items.

The first component (4 items, explained variance = 37.23%) was loaded by items referred to perceived stress related to relationships with relatives, relationships with colleagues, relationships with professors, and academic studying. We labeled this scale Relationships and Academic Life.

The second component (2 items, explained variance = 17.20%) was loaded by items referred to perceived stress related to social isolation and changes in couples’ relationship, intimacy and sexual life due to the social isolation. We labeled this scale Isolation.

The third component (1 item, explained variance = 12.66%) was loaded by a single item referred to perceived stress related to the risk of infection, hence it was labeled as Fear of Contagion (Table 3).

**TABLE 3:** COVID-19 Student Stress Questionnaire (CSSQ) exploratory factor analysis on first random split-half sample (*n* = 257).

Factors and Items	1	2	3	*h*^2^
Factor 1: Relationships and Academic Life				
4. How do you perceive the relationships with your university colleagues during this period of COVID-19 pandemic?	**0.904**	−0.419	0.109	0.732
5. How do you perceive the relationships with your university professors during this period of COVID-19 pandemic?	**0.687**	0.240	−0.136	0.646
6. How do you perceive your academic studying experience during this period of COVID-19 pandemic?	**0.560**	0.381	−0.207	0.621
3. How do you perceive the relationships with your relatives during this period of COVID-19 pandemic?	**0.491**	0.180	0.271	0.441
Factor 2: Isolation				
7. How do you perceive the changes in your sexual life due to the social isolation during this period of COVID-19 pandemic?	−0.139	**0.838**	0.048	0.650
2. How do you perceive the condition of social isolation imposed during this period of COVID-19 pandemic?	0.088	**0.788**	0.128	0.722
Factor 3: Fear of Contagion				
1. How do you perceive the risk of contagion during this period of COVID-19 pandemic?	−0.003	0.122	**0.917**	0.885
Eigenvalue	2.61	1.20	1.00	
Percentage of variance	37.23	17.20	12.66	

*Total variance explained = 67.09%. Cronbach’s α = 0.71. Values in bold indicate major loadings. *h*^2^ is item communality.*

#### Confirmatory Factor Analysis

**FIGURE 1:**
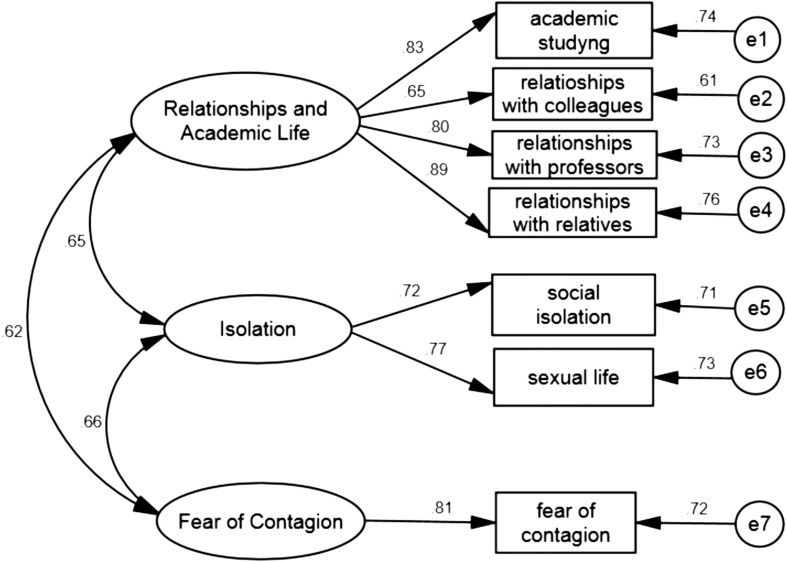
Path diagram and estimates for the three-factor COVID-19 Student Stress Questionnaire on second random split-half sample (*n* = 257).

Confirmatory Factors Analysis (CFA) was run to test hypothesis 2. The results supported the PCA findings (Figure 1) by demonstrating that the three-factors model (χ^2^ = 4.52, *p* = 0.79), comprising all the 7 items proposed, yielded good fit for all of indices (χ*^2^*/df ratio = 0.56; CFI = 0.95; TLI = 0.95; RMSEA = 0.06).

### Convergent and Discriminant Validity

Concerning Convergent validity, the standardized factor loadings of CSSQ items were all > 0.5 (see Figure 1) and statistically significant (*p* < 0.001). Moreover, the CR values were all > 0.7 (i.e., Relationships and Academic Life CR = 0.924; Isolation CR = 0.809; Fear of Contagion CR = 0.769). The values of AVE of all factors were > 0.5 (i.e., Relationships and Academic Life AVE = 0.637; Isolation AVE = 0.549; Fear of Contagion AVE = 0.649). Therefore, the standardized factor loadings, CR and AVE of factors were united to suggest that the CSSQ had strong convergent validity, confirming hypothesis 3.

Moreover, correlations with measures of psychophysical disease (SCL-90-R subscales and GSI) were carried out to further test convergent validity, showing that COVID-19 Student Stress Questionnaire scales and Global Stress scores revealed moderate to strong correlations with the SCL-90-R scales scores in the expected directions, and confirming hypothesis 4 (Table 4).

**TABLE 4:** Correlations of the COVID-19 Student Stress Questionnaire (CSSQ) scales with SCL-90-R scales.

	COVID-19 Student Stress Questionnaire Scales			
	
SCL-90-R Scales	Relationships and Academic Life	Isolation	Fear of Contagion	Global Stress
Anxiety	0.450**	0.337**	0.533**	0.552**
Depression	0.494**	0.386**	0.393**	0.565**
Somatization	0.312**	0.313**	0.275**	0.393**
Obsessive-Compulsive	0.490**	0.262**	0.353**	0.500**
Interpersonal Sensitivity	0.457**	0.266**	0.378**	0.487**
Hostility	0.481**	0.354**	0.346**	0.532**
Phobic Anxiety	0.352**	0.154*	0.495**	0.405**
Paranoid Ideation	0.411**	0.309**	0.292**	0.456**
Psychoticism	0.372**	0.313**	0.428**	0.467**
Global Severity Index (GSI)	0.545**	0.405**	0.475**	0.624**

*p < 0.05; **p < 0.01.

Concerning Discriminant validity, the square root of AVE values were compared with the correlations among factors. All the square root of AVE values (i.e., Relationships and Academic Life, SQRT AVE = 0.798; Isolation SQRT AVE = 0.741; Fear of Contagion SQRT AVE = 0.805) were above the correlation values (i.e., correlation between Relationships and Academic Life and Isolation, *r* = 0.645; correlation between Relationships and Academic Life and Fear of Contagion, *r* = 0.621; correlation between Isolation and Fear of Contagion, *r* = 0.660; see Figure 1), indicating suitable discriminant validity, and supporting hypothesis 5.

Furthermore, still concerning discriminant validity, intercorrelations between the three COVID-19 Student Stress Questionnaire scales and the Global Stress scores were also calculated. Intercorrelations ranged from 0.30 to 0.42, showing medium levels of correlation, while correlations of all COVID-19 Student Stress Questionnaire scales with Global Stress scores were high in size and significant, indicating that the questionnaire assessed different but related dimensions, and confirming hypothesis 6 (Table 5).

**TABLE 5:** Intercorrelations between the COVID-19 Student Stress Questionnaire (CSSQ) scales.

CSSQ scales	Relationships and Academic Life	Isolation	Fear of Contagion	Global Stress
Relationships and Academic Life	1			
Isolation	0.417**	1		
Fear of Contagion	0.344**	0.298**	1	
Global Stress	0.871**	0.757**	0.587**	1

*p < 0.05; **p < 0.01.

### Item Analysis and Reliability

Mean scores for the single items varied from a maximum score of 2.01 (Item 2: “How do you perceive the condition of social isolation imposed during this period of COVID-19 pandemic?”) to a minimum of 0.44 (Item 4: “How do you perceive the relationships with your university colleagues during this period of COVID-19 pandemic?”). SDs for the single items varied from 1.36 (Item 7: “How do you perceive the changes in your sexual life due to the social isolation during this period of COVID-19 pandemic?”) to 0.75 (Item 4: “How do you perceive the relationships with your university colleagues during this period of COVID-19 pandemic?”). The mean inter-item correlation was 0.26, therefore it was satisfactory. Cronbach’s alpha of the total scale was 0.71, while McDonald’s omega coefficient was 0.71, confirming that the CSSQ had satisfactory internal consistency (hypothesis 7).

All the items of the CSSQ were presented in Table 6.

**TABLE 6:** The COVID-19 Student Stress Questionnaire.

	Not at all Stressful	Somewhat stressful	Moderately Stressful	Very Stressful	Extremely Stressful
1. How do you perceive the risk of contagion during this period of COVID-19 pandemic?	0	1	2	3	4
(Come vive il rischio di contagio durante l’attuale periodo di pandemia COVID-19?)					
2. How do you perceive the condition of social isolation imposed during this period of COVID-19 pandemic?	0	1	2	3	4
(Come vive la condizione di isolamento sociale imposta durante l’attuale periodo di pandemia COVID-19?)					
3. How do you perceive the relatioships with your relatives during this period of COVID-19 pandemic?	0	1	2	3	4
(Come vive le relazioni con i suoi familiari durante l’attuale periodo di pandemia COVID-19?)					
4. How do you perceive the relationships with your university colleagues during this period of COVID-19 pandemic?	0	1	2	3	4
(Come vive il suo rapporto con i colleghi universitari durante l’attuale periodo di pandemia COVID-19?)					
5. How do you perceive the relationships with your university professors during this period of COVID-19 pandemic?	0	1	2	3	4
(Come vive il suo rapporto con i docenti universitari durante l’attuale periodo di pandemia COVID-19?)					
6. How do you perceive your academic studying experience during this period of COVID-19 pandemic?	0	1	2	3	4
(Come vive la sua esperienza di studio universitario durante l’attuale periodo di pandemia COVID-19?)					
7. How do you perceive the changes in your sexual life due to the social isolation during this period of COVID-19 pandemic?	0	1	2	3	4
(Come vive i cambiamenti nella sua vita sessuale causati dall’isolamento durante l’attuale periodo di pandemia COVID-19?)					
	_____ +	_____ +	_____ +	_____ +	_____ +
	Global Score _______				

*The Italian version is provided in brackets.*

Table 7 displays items, means, standard deviations, and ranges of the CSSQ scales (Relationships and Academic Life, Isolation, Fear of Contagion) and the total score (Global Stress). Considering that high levels of COVID-19-related stress can be indicated by scores that are 1 SD above the mean (e.g., the 84th percentile) and low levels of stress can be indicated by scores that are 1 SD below the mean (e.g., the 16th percentile) of the distribution of the CSSQ scores, we can affirm that scores of 6 or below indicate low levels of perceived COVID-19-related Global stress, scores of 7–15 indicate average levels of perceived COVID-19-related Global stress, and scores of 16 or more indicate high levels of perceived COVID-19-related Global stress among university students.

**TABLE 7:** Items, mean, SD and range scores of the COVID-19 Student Stress Questionnaire scales.

CSSQ Scales	Items	Mean ± SD	Range
Relationships and Academic Life	3, 4, 5, 6	4.95 ± 2.74	0–13
Isolation	2, 7	3.51 ± 2.05	0–8
Fear of Contagion	1	1.61 ± 1.12	0–4
Global Stress	All items	10.07 ± 4.52	1–22

## Discussion

The aim of the present study was to develop, validate and evaluate the psychometric properties of the 7-item COVID-19 Student Stress Questionnaire (CSSQ), a brief measure to assess sources of stress related to the COVID-19 pandemic lockdown among university students. Indeed, addressing specific sources of stress tailored to target populations foster efficacy in preventive efforts and interventions (Zurlo et al., 2013, 2017; Anniko et al., 2019).

Accordingly, responding to the widespread need for developing specific tools to understand the impact of the COVID-19 global pandemic among students (Cao et al., 2020; Lee, 2020b; Sahu, 2020), it was hoped this instrument could foster a timely identification of those students at higher risk for developing a significant disease related to the ongoing unique situation, and to deliver evidence-based and tailored interventions to promote their adjustment and wellbeing.

Findings highlighted that the proposed CSSQ possessed adequate factor validity, tapping three meaningful factors.

The first factor, labeled Relationships and Academic Life, comprised four items covering perceived stress related to relationships with relatives, relationships with colleagues, relationships with professors, and academic studying. Indeed, considering that students’ daily routine have been subject to specific changes (Cao et al., 2020; Chen et al., 2020; Lee, 2020b), this first factor fostered a greater understanding of the dimensions characterizing these modifications among university students in terms of relationships and academic life.

From this perspective, the relationships with relatives should be carefully focused, considering the forced full-time cohabitation, with almost exclusive sharing time and spaces throughout all days. This also as a consequence of the closures of the campus and students accommodations, which forced several students to return back home, but also considering the great number of students already living with their parents, however under completely changed conditions.

In the same direction, since restrictions drastically impaired the possibilities to benefit from living the university life, university students may report growing disease connected to changes in relationships with colleagues and professors (that, during the COVID-19 pandemic, are only allowed through online platforms), but also increased suffering related to the academic studying (e.g., fear of delays, difficulties in finding appropriate spaces to concentrate) (Cao et al., 2020; Lee, 2020b; Sahu, 2020).

The second factor, labeled Isolation, comprised two items exploring perceived stress related to social isolation and changes in sexual life due to the containment measures. From this perspective, in line with research emphasizing the strong weight of containment measures such as quarantine and social distancing on individuals’ psychological health and wellbeing (Brooks et al., 2020; Horesh and Brown, 2020; Lee, 2020a; Williams et al., 2020), the second factor also captured the perceived disease and sense of loneliness derived from living this condition, often far from the loved ones (Sahu, 2020; Zhai and Du, 2020).

From this perspective, considering the specificity of the target population, it’s not surprising that the confinement in itself and sexual life belonged to the same factor. Indeed, since students were more likely to still live with their families or they returned back home due to the pandemic, it’s more probable that their couple’ relationship, intimacy and sexual life were subject to significant restrictions due to the lockdown. However, these findings may be also due to the specific European context, considering that the average age of young people leaving the parental house is 25.9 (Eurostat, 2020), while in several other countries students use to leave home around 18 years for starting the college (Aassve et al., 2002; Crocetti and Meeus, 2014).

The third factor, labeled Fear of Contagion, comprised one item assessing perceived stress related to the risk of infection. The relevance of the latter dimension is, indeed, in line with previous studies on the key role played by the fear to be infected, the fear for others (e.g., relatives, friends) to become ill, as well as the fear to be a source of contagion for the others (Ahorsu et al., 2020; Brooks et al., 2020; Taylor et al., 2020).

Concerning convergent validity, the standardized factor loadings, and the values of AVE and CR were well above the threshold suggested by Hair et al. (2010), indicating that the variances were more explained by each factor and all of the items of each factor were consistent for measuring the same latent construct.

Furthermore, data revealed significant associations of all CSSQ scales scores with all the SCL-90-R standardized scales scores as well as with the Global Severity Index. This revealed how the specific sources of stress we have identified, covering changes in Relationships and Academic Life, perceived Isolation and Fear of Contagion, could have significant negative effects on perceived psychophysical health conditions among students. These results suggested the meaningfulness to adopt the proposed instrument also to foster the development of early interventions supporting students’ adjustment and promoting their psychophysical health during and after the COVID-19 pandemic lockdown.

Concerning discriminant validity, the square root of AVE values were greater than the correlations coefficients between the factors, indicating that the three factors could extract more variance than the sharing among factors, so revealing a satisfactory discriminant validity. Moreover, intercorrelations between COVID-19 Student Stress Questionnaire scales (moderate in size) and correlations between the three scales and the Global Stress score (high in size) confirmed that the CSSQ assessed different but connected dimensions, so giving further support about the validity of the proposed tool to evaluate both perceived Global Stress and different sources of stress related to the COVID-19 pandemic. Therefore, both perceived levels of Global Stress and specific stressors should be carefully considered when defining interventions fostering students’ wellbeing during the current COVID-19 crisis.

Finally, the evaluation of mean inter-item correlation, Cronbach’s alpha and McDonald’s omega confirmed that the CSSQ had satisfactory internal consistency.

In conclusion, this study demonstrated that the COVID-19 Student Stress Questionnaire is a 7-item multidimensional scale with satisfactory psychometric properties. Moreover, it is a good instrument to be used in assessing and allaying perceived COVID-19-related stress among university students.

### Implications for Clinical Practice

The study sought to address the growing concerns arising from the challenges that students around the world are facing due to the COVID-19 pandemic and from its potential negative effects on their psychophysical health conditions, by providing a brief, valid and meaningful tool, namely the COVID-19 Student Stress Questionnaire (CSSQ).

The CSSQ presented here is a brief multidimensional tool, conceived to be helpfully used by members from different areas within universities (e.g., human resources, health units, student affairs) to promote a deeper understanding of the nature of COVID-19-related stressors perceived by students, in order to define tailored policies and support interventions.

In line with this, the CSSQ could be useful to early identify those students in need of psychological support. Indeed, due to the perceived risk of contagion, the consequent modifications of all significant relationships in daily life may induce, among university students, loss of contact with formal and informal support networks and growing risk of isolation. Therefore, it becomes pivotal to make all the possible efforts to assure careful monitoring of their perceived levels of stress and psychological wellbeing.

Finally, the adoption of the CSSQ in the clinical practice can significantly help social and health practitioners, serving as a monitoring and evaluation tool to define more tailored evidence-based counseling interventions. Indeed, since tapping different stressors that could have been experienced due to the COVID-19 outbreak (i.e., stressors related to Relationships and Academic Life, Isolation, and Fear of Contagion), the adoption of this tool can help to underline those areas requiring more attention within counseling interventions and to assess the effectiveness of the interventions by evaluating potential changes over time.

### Limitations and Future Research

Despite these strengthens, some limitations need to be underlined. Firstly, the administering of the questionnaire was online, potentially limiting the enrollment in the study of those without Internet access. However, since the target population of Italian university students (taking into account both the age and the provision of distance learning during the COVID-19 pandemic), we consider this limitation could have influenced our results to a little extent. Secondly, the participant pool comprised a self-selected sample of students enrolled only in one university (i.e., students enrolled in Philosophy, Modern Languages and Literature, and Psychology degree courses) with a majority being female (and therefore, tests for gender differences were not possible). Further investigation on bigger and more representative samples is needed to confirm the results provided by the present study (e.g., a nationally representative sample with more male participants). Thirdly, the study relies on participants’ self-reports, and, therefore, findings could be affected by the risk of social desirability bias. Future research could, hence, include a broader range of sources of data. Furthermore, future studies could also consider the meaningfulness to adopt newly developed COVID-19-related instruments (e.g., FCV-19S) to test concurrent validity. Indeed, at the time of study design and data collection, the Italian versions of these specific measurement tools were not available yet. Another limitation is the lack of available data for a more robust examination of reliability beyond internal consistency, such as test-retest. Consequently, future studies could be designed with the aim to also conduct test-retest analysis. Finally, cultural and social variables may have potentially influenced the construct of the questionnaire as well as it’s convergent and discriminant validity. Consequently, further applications of this instrument in other countries are needed to allow gaining further information about sources of stress influencing students’ wellbeing according to different countries worldwide.

Notwithstanding these limitations, this study provided researchers and practitioners with a brief, easily administered, valid and reliable measure to assess perceived stress among university students, so supporting efforts to understand the impact of this unique global crisis and develop tailored interventions fostering students’ wellbeing.

## Data Availability Statement

The raw data supporting the conclusions of this article will be made available by the authors, without undue reservation.

## Ethics Statement

The studies involving human participants were reviewed and approved by Ethical Committee of Psychological Research of the University of Naples Federico II. The patients/participants provided their written informed consent to participate in this study.

## Author Contributions

MCZ: study conception and design, interpretation of data, drafting of manuscript, critical revision. MFCDV: analysis and interpretation of data, and drafting of manuscript. FV: acquisition of data, analysis and interpretation of data, and drafting of manuscript. All authors read and approved the final manuscript.

## Conflict of Interest

The authors declare that the research was conducted in the absence of any commercial or financial relationships that could be construed as a potential conflict of interest.
